# Physician Suicide in the Era of the COVID-19 Pandemic

**DOI:** 10.7759/cureus.19313

**Published:** 2021-11-06

**Authors:** Christopher W Laboe, Ankit Jain, Krishna Priya Bodicherla, Meenal Pathak

**Affiliations:** 1 Psychiatry & Behavioral Health, Penn State College of Medicine, Hershey, USA; 2 Psychiatry, Manhattan Psychiatric Center, New York, USA

**Keywords:** resident well-being, depression in a physician, covid-19, professional burnout, physician suicide

## Abstract

Physician burnout is a common problem among US physicians. Burnout has been associated with absenteeism, mood disorders, and medical errors. Over the last several decades, physician burnout has become more prevalent because of increasing workloads, increasing administrative burden, and time spent on electronic medical records, among several other reasons. The rate of suicidal ideation in physicians is almost twice as high as the general population. In addition, studies on mortality related to suicide show that the rates of suicides in physicians are consistently higher than in the general population. Firearms are the most common suicide method in both groups, while physicians are more likely to use poisoning and blunt force trauma, as physicians who committed suicide were more likely to have benzodiazepines, barbiturates, or antipsychotics detectable in their blood. Unfortunately, coronavirus disease 2019 (COVID-19) brought to the surface multiple prevailing issues in the US healthcare system, including physician burnout and the prevalence of suicidality among physicians in the recent past. With this editorial, we plan to discuss the current understanding of the impact on physician suicide in the context of COVID-19.

## Editorial

Coronavirus disease 2019 (COVID-19) has caused more than 80 million confirmed infections and more than 1.8 million people had died by the end of 2020. Globally, there have been 242,348,657 confirmed cases of COVID-19, including 4,927,723 deaths, reported to the World Health Organization. As of October 21, 2021, a total of 6,655,399,359 vaccine doses have been administered. COVID-19 led to social distancing and isolation, and there has been evidence of its ill effects on pre-existing mental health issues and the development of new mental health issues [1,2]. Specifically, it has been reported that social distancing and isolation resulting in loneliness worsen the symptoms of anxiety and depression [1,3]. Masking during the COVID-19 pandemic has led to decompensation of multiple psychiatric comorbidities, including depression, anxiety, and post-traumatic stress disorder symptoms in some individuals [4]. Excessive cleaning and recommended hygiene practices during COVID-19 have potentially contributed to the worsening symptoms of obsessive-compulsive disorder in many individuals [5]. There has also been a growing body of literature since the beginning of the pandemic on the neuropsychiatric complications of COVID-19 infection [6]. Overall, the COVID-19 pandemic has derailed multiple aspects of an individual’s life, and multiple stressors have emerged because of the effects of the pandemic. The increased stressors due to the COVID-19 pandemic on the family unit have led to increasing mental health concerns across the lifespan from parents to their children [7,8]. In addition to the detrimental effect on the general public’s mental health, it has been shown that frontline healthcare workers experienced significant distress during COVID-19 [9]. Noteworthy factors in the development of distress in healthcare workers included feelings of loss of control and vulnerability and difficulty adapting to change [10]. Due to the increased rates of burnout among healthcare workers, vicarious trauma, and increased stress from social isolation, an increase in physician suicides rates is expected. With this editorial, we plan to discuss the current understanding of the impact on physician suicide in the context of COVID-19.

Over the last several decades, physician burnout has become more prevalent because of increasing workloads, increasing administrative burden, and time spent on electronic medical records, among several other reasons [11]. The rate of suicidal ideation among physicians is almost twice as high as the general population at 7.2% vs. 4%. In addition, studies on mortality related to suicide show that the rates of suicides in physicians are consistently higher than in the general population [12]. Suicide is the only cause of mortality that is higher in physicians compared to nonphysicians. Compared with nonphysicians, male physicians are 40% more likely to die by suicide, and the risk to female physicians is more than doubled [13]. Firearms are the most common suicide method in both groups, while physicians are more likely to use poisoning and blunt force trauma, as physicians who committed suicide were more likely to have benzodiazepines, barbiturates, or antipsychotics detectable in their blood [14]. Unfortunately, COVID-19 brought to the surface multiple prevailing issues in the US healthcare system, including physician burnout and the prevalence of suicidality among physicians [11,14]. It is likely that the emotional distress stemming from the increased patient burden, longer duty hours, poor physician coverage pool, the ever-looming threat of contracting COVID-19 and getting severely ill, and staying away from family and loved ones to ensure social isolation has potentially led to the worsening of mental health and, thus, contributing to an increase in physician suicide during the COVID-19 pandemic. To that effect, there have been many cases of physician suicide during COVID-19 that have been recently reported in the media. One such case is of a physician in New York City, Dr. Breen, who experienced excessive exhaustion putting in long hours at work in the emergency room [15]. She contracted COVID-19 and took 1.5 weeks off to recover, but upon returning, she was incapable of handling her previous workload due to exhaustion [15]. After a brief return to her job, on a friend’s advice, she went to stay with family in Charlottesville, VA, and then was admitted to the UVA hospital to return to family in Virginia. She was treated for exhaustion for about a week before returning to her mom’s house in Virginia [15]. The following week she was found in her sister’s house after an attempted suicide and was taken to UVA’s hospital where she succumbed to self-inflicted wounds [15]. Another noteworthy issue in physician burnout contributing to worsening suicide is a low emphasis on resident physician wellness, especially during the pandemic, who often work long hours and stay away from their families. A considerable number of physicians in the US primary care specialties consists of International Medical Graduates (IMGs), many of whom have not visited their home countries in the context of US embassy closures and travel restrictions [14,16]. Medical students applying for residency and matching process are also encountering challenges with the changes and experiencing burnout [17].

There may be an expectation in our society that physicians should be putting their patients first before themselves, both in terms of their physical and mental health needs. The social support of physicians grappling with psychiatric issues is not as readily available as it should be, even though there may be resources that they can use. Our society needs more guidance to professional social support services as soon as issues arise at work without fear of stigma and retaliation.

Some factors that increase burnout in the workplace, leading to increased suicide risk, are control and flexibility of work, meaning in work, the community at work, the flow of work, and resource availability [18]. If there is an issue in one of these areas, physicians should report to the administration staff, and the administration should address it through data gathering and problem-solving. It has been suggested that giving individuals more control of their time and schedule is a great starting point toward decreasing burnout and physician suicide. More resources should be channeled to help physicians recover from vicarious trauma that they experience to prevent physician suicide.

Conclusions

Physician wellness has long been a challenging area to address but a very important issue during the COVID-19 pandemic. There is a need for more discussion regarding setting up peer support groups at the workplace. Crisis hotlines are another option that might have been an underutilized resource. More efforts need to be placed on being able to take time off for physical or mental health appointments during busy work schedules. Peer support groups among physicians regarding COVID-related stress including increased workload as well as the ever-looming fear of contracting COVID-19 and developing complications secondary to COVID-19 infection need to be established. There have been many physicians and healthcare staff deaths from COVID-19 infections and many others who required prolonged hospitalization and developed severe complications from it. Physicians and trainees should be able to access mental health support groups in this context. While vaccinations are helping reduce the infection rates and help boost the morale of healthcare staff including physicians, there needs to be increased emphasis on providing much-needed support for their mental well-being. Physician burnout is a common problem among US physicians. Burnout has been associated with absenteeism, mood disorders, and medical errors. Because of its prevalence and effects on professionalism, access, and quality of care, the issue of physician burnout should be discussed, along with how to effectively reform the healthcare system. Despite the potentially serious consequences of burnout, there are few interventions designed to combat this problem. For reform to achieve its goal of providing all physicians access to high-quality medical care, efforts to identify and address the controllable factors contributing to burnout among physicians are needed. Doing so is vital for patients to receive compassionate care from committed, competent, and professional physicians.
